# A general method for the unbiased improvement of solution NMR structures by the use of related X-Ray data, the AUREMOL-ISIC algorithm

**DOI:** 10.1186/1472-6807-6-14

**Published:** 2006-06-26

**Authors:** Konrad Brunner, Wolfram Gronwald, Jochen M Trenner, Klaus-Peter Neidig, Hans Robert Kalbitzer

**Affiliations:** 1 Department of Biophysics and Physical Biochemistry, University of Regensburg, Postfach, D-93040 Regensburg, Federal Republic of Germany; 2 Bruker BioSpin GmbH, Software Department, Silberstreifen 4, D-76287 Rheinstetten, Federal Republic of Germany

## Abstract

**Background:**

Rapid and accurate three-dimensional structure determination of biological macromolecules is mandatory to keep up with the vast progress made in the identification of primary sequence information. During the last few years the amount of data deposited in the protein data bank has substantially increased providing additional information for novel structure determination projects. The key question is how to combine the available database information with the experimental data of the current project ensuring that only relevant information is used and a correct structural bias is produced. For this purpose a novel fully automated algorithm based on Bayesian reasoning has been developed. It allows the combination of structural information from different sources in a consistent way to obtain high quality structures with a limited set of experimental data. The new ISIC (*I*ntelligent *S*tructural *I*nformation *C*ombination) algorithm is part of the larger AUREMOL software package.

**Results:**

Our new approach was successfully tested on the improvement of the solution NMR structures of the Ras-binding domain of Byr2 from *Schizosaccharomyces pombe*, the Ras-binding domain of RalGDS from *human *calculated from a limited set of NMR data, and the immunoglobulin binding domain from protein G from *Streptococcus *by their corresponding X-ray structures. In all test cases clearly improved structures were obtained. The largest danger in using data from other sources is a possible bias towards the added structure. In the worst case instead of a refined target structure the structure from the additional source is essentially reproduced. We could clearly show that the ISIC algorithm treats these difficulties properly.

**Conclusion:**

In summary, we present a novel fully automated method to combine strongly coupled knowledge from different sources. The combination with validation tools such as the calculation of NMR *R*-factors strengthens the impact of the method considerably since the improvement of the structures can be assessed quantitatively. The ISIC method can be applied to a large number of similar problems where the quality of the obtained three-dimensional structures is limited by the available experimental data like the improvement of large NMR structures calculated from sparse experimental data or the refinement of low resolution X-ray structures. Also structures may be refined using other available structural information such as homology models.

## Background

In any structure determination process of a biological macromolecule the general goal is to obtain from the available data a structure as accurate as possible. For all high throughput procedures as used in structural genomics projects the structure determination process has to be as fast as possible, demanding that only a minimal set of experimental data is recorded. One way to speed up the NMR structure determination process is to reduce the required number of experimental restraints and/or to use only restraints that are relatively easy to obtain e.g. backbone dihedral angles, chemical shifts, residual dipolar couplings, hydrogen bonds, or H^N^-H^N ^NOEs. When the amount of available experimental data is limited, the use of additional information such as structural data from homologous proteins is advisable. Most fast methods previously described in the literature are mainly aimed at determining the global fold of a protein [1-9]. Another set of methods directly uses information from different sources, namely NMR and X-ray, for joint structure refinement to obtain refined structures. It is common to these approaches that discrepancies between NMR and X-ray data are manually corrected, for example by removing violated NOEs, reassigning NOEs or hydrogen-bonds, and taking spin-diffusion effects on NMR restraints into account [10-15].

From the conceptual point of view in any structural prediction or calculation from a set of mixed data one has to decide beforehand what kind of structure is the target of the procedure since there is nothing like "the structure". This question is inherently answered in purely experimental structure determination since solution NMR spectroscopy determines the structure in solution and a crystal structure in the crystal. More importantly, the selected experimental conditions such as the buffer and the absence or presence of ligands select the target structural set.

Here, we present a novel general and fully automated approach called ISIC (*I*ntelligent *S*tructural *I*nformation *C*ombination) for the combination of structural information from different sources. It allows the predefinition and selection of the target structural set and properly treats discrepancies inherent in the input structural data, thereby ensuring that the additional input data are properly biased toward the target structural set. Using the combined information, high resolution structures are calculated and results are automatically verified on experimental data. One possible application of the ISIC algorithm for rapid structure determination would include the use experimental solution NMR data that is relatively easy to obtain, such as backbone dihedral angles, chemical shifts, residual dipolar couplings, hydrogen bonds, or H^N^-H^N ^NOEs that alone allow the calculation of a low to medium resolution NMR structure, supplemented with for example data from homology modeling or from a homologous X-ray structure.

In this paper, ISIC was tested for three applications that may occur in "real life". Firstly, the refinement of a solution structure of a protein with an X-ray structure of the same protein determined under slightly different conditions (proper choice), secondly the refinement of a structure calculated from a limited set of NMR data with an X-ray structure of the same protein also determined under slightly different conditions and last, the refinement of a known NMR structure with a known X-ray structure of the same protein that is largely different (wrong choice). For the first case we selected the Ras-binding domain of Byr2 (Byr2-RBD) from *Schizosaccharomyces pombe *(residues 71–165 referred here as residues 1–95) for which both a solution structure of the free protein [16] and a crystal structure of Byr2-RBD in complex with Ras [17] are available. Both structures are of medium quality of about 3 Å resolution (X-ray) or equivalent resolution (NMR) making it an ideal target for structure refinement. In addition, it is expected that the two structures are not identical since complex formation with Ras leads to small but significant conformational changes in the structure of Byr2. The aim of the second test was to refine a structure that was obtained using only readily available NMR data. For this case the Ras-binding domain of RalGDS (RalGDS-RBD) from *human *was used. The solution structure (residues 1–97, corresponding to residues 788–884 of the full length protein, Swiss prot accession code: Q12967) has been published previously [18]. For the current tests the low resolution structure of a shorter construct (amino acid 11 to 97) was obtained by using only relatively easily available NMR data such as h-bonds, dihedral angles, and back-bone NOEs. In addition a medium quality (3.4 Å resolution) X-ray structure of RalGDS in complex with Ras is available [19]. Similar to the first test case small but significant conformational changes between RalGDS in its free solution form and its crystal form in complex with Ras are expected. As a third example we used the NMR [20] [PDBID:1Q10] and the crystal structure [21] [PDB ID: 1PGX] of the immunoglobulin binding domain of protein G from *Streptococcus*, *species Lancefield group G*. In this case large global structural differences were observed since in solution dimerization introduced by core mutations induces a domain swapping of a β-pleated sheet.

## Results

### Theoretical considerations

#### General considerations

In the improvement of structures by including information from other sources two main cases have to be distinguished: In the first case the additional information is describing the same set of structures (e. g. a solution structure of a protein at given pH, temperature and sample composition). Here the proper weighting of the additional information is the main point when the "true" structure should be optimally approximated. In the second case the additional information is taken from structures that are supposed to be similar but are different nevertheless (e. g. a solution structure and a crystal structure of a different complex). Here an additional difficulty arises since one has to estimate how well the additional structure will apply to the structure in question since otherwise not a properly biased solution will be obtained. The problem can be formulated as the aim to obtain the most probable structure or the most probable set of structures *S*_0 _with a conditional probability *P*(*S*_0_|*A*, *I*_i_, i = 1, N) higher than a threshold value *P*_t_. The combination of information from *N *different sources *I*_i _is a problem often encountered in structural biology. When *S*_0 _is a set of purely NMR derived protein structures, *A *would be the general knowledge about the system that is the physical model including the covalent structure and the interaction potentials as they enter a typical molecular dynamics calculation. The NMR derived information *I*_1 _is usually expressed as a set of experimental restraints *R*_1 _= {*R*_1_^1^,...., *R*_1_^M^} containing *M *restraints that essentially reduce the accessible conformational space of the probable solutions. The experimental restraints are rather inhomogeneous since they include information such as distance restraints from NOESY spectra, dihedral angle information from J-couplings or chemical shifts, as well as intra molecular orientational restraints from residual dipolar couplings.

An elegant semi quantitative way to find the most probable structures *S*_i _is the simulated annealing protocol [22], where the information *A *is an intrinsic part of the molecular dynamics routines used.

In case two the situation becomes much more complex since structural information that corresponds not exactly to the conditions used in the actual experiment is added from other sources. When this information is expressed again in the form of sets of restraints *R*_i_, structures *S*_0_^p ^(p = 1,...,L_0_, with L_0 _being the total number of structures in set S_0_) have to be found with high probabilities *P*(*S*_0_^p ^| *A*, *R*_i. _i = 1,...,N). When a restrained simulated annealing approach is used, the physical model is again an implicit feature, that is *P*(*S*_0_^p ^| *A*, *R*_i. _i = 1,...,N) can be replaced by *P*(*S*_0_^p ^|*R*_i. _i = 1,...,N). With the exception of the restraint set *R*_1 _corresponding to the leading set of structures *S*_1_, the primary restraints *R*_i_* (i = 2,...,N) that are derived from the other sources in general do not directly apply to the conditions of the leading set of structures. This can for example occur due to different experimental conditions. As a consequence, new restraints *R*_i _have to be calculated, which directly apply to the true set of structures *S*_0_. This means that for *R*_1 _one can define *R*_1 _= *R*_1_*, but for the other restraint sets *R*_i_* we have to determine to which amount their individual restraints apply to the true structures *S*_0_, as explained below.

*P*(*S*_0_|*R*_i. _i = 1,...,N) = *P*(*S*_i_|*R*_1_* = *R*_1_, *R*_i_*, i = 2,...,N)     (1)

In general, the complete description of the sets of restraints *R*_i _has to be given as a multidimensional probability distribution *p*(*R*_i_, i = 1,...,N). The different sets of restraints and the restraints themselves are coupled since they are derived from related structures and coupled by the physical model. The probability *P *and thus the probability distribution *p *of a set of restraints *R*_i _in the leading structures can be calculated from the known *R*_i_* by

*P*(*R*_i_) = *P*(*R*_i_|*R*_i_*, i = 1,...,N)*P*(*R*_i_*, i = 1,...,N)     (2)

Equation 2 shows that *R*_i _depends again on a multidimensional probability distribution and a simplification of the problem is mandatory.

In the standard simulated annealing approach the individual restraints *R*_i_^k ^are assumed primarily as independent, their coupling is performed indirectly by the algorithm itself, which selects consistent solutions. As long as the same restraints *R*_i_^k ^are considered (and the restraints in a given structure can be considered to be uncoupled) one can calculate the probability that a newly created restraint *R*_0_^k ^that corresponds to the "true" solution structures *S*_0 _has a given value in the set *S*_0_. The restraints *R*_0_^k ^are used later on for calculating the set of true solution structures *S*_0_.

*P*(*R*_0_^k^) = *P*(*R*_0_^k^|*R*_i_^k*^, i = 1,...,N)*P*(*R*_i_^k*^, i = 1,...,N)     (3)

The indices i and k specify the data set used and the specific restraint, respectively. Here, it is assumed that in first order the individual restraints *R*_0_^k ^and *R*_0_^l ^are independent for k≠l. For the calculation of *P*(*R*_0_^k^) it would be useful to have information about the same restraints in the structures derived from the different data sets. Below it will be shown how a reasonable estimate can be obtained by using a MD-sampling procedure.

Equation 3 can be used in two different ways: When a good estimate of the conditional probability is known it can be directly applied. If this is not the case, one can test the hypothesis that *P*(*R*_0_^k^|*R*_i_^k^*) is close to 1 for a data set i. Since we assume that the experimental data 1 represents the "true" ensemble, one can test if a restraint *R*_i_^k ^is part of the same ensemble as *R*_1_^k ^and simply discard all restraints *R*_i_^k ^in the calculation that do not fulfill the condition. *P*(*R*_i_^k*^, i = 1,...,N) in eq. 3 describes the probability that a substitute restraint *R*_i_^k* ^has a given value in the set of structures *S*_i _and clearly this probability depends on factors such as the corresponding second moments σ of the restraints in the set of structures *S*_i_.

#### Main features of the algorithm

**Figure 1:**
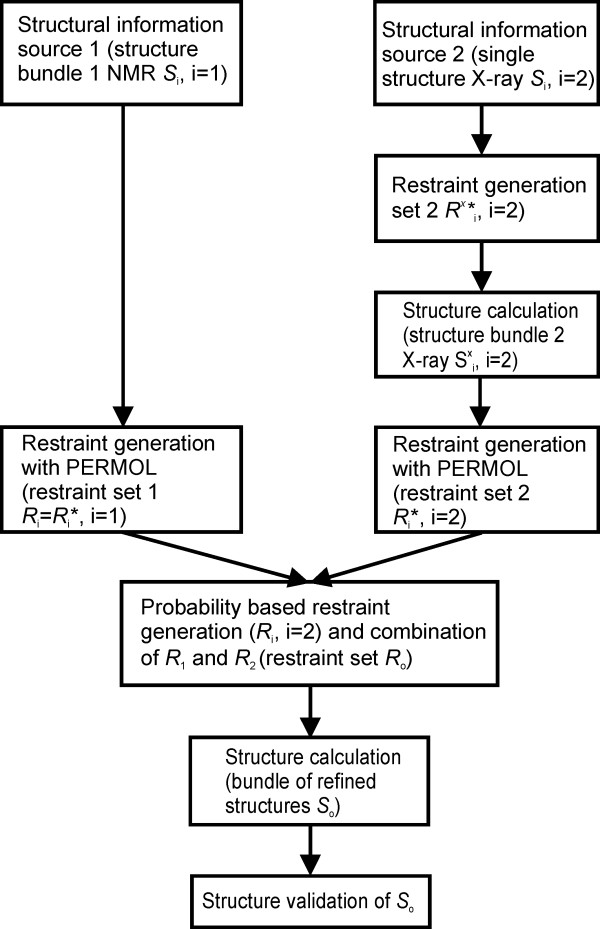
**Schematic description of the ISIC algorithm**. In the above example two input sources are used, one representing a bundle of NMR structures *S*_1 _and one representing a single X-ray structure *S*_2_.

The general features of the ISIC algorithm based on the above considerations are described in Figure 1 for the important application that a NMR solution structure is improved by an X-ray-structure. In ISIC the structural information from a set of different sources i consisting of members *S*_i _(with i = 1,...,N and the number of used sources N ≥ 2) is used to improve the structures of the set *S*_1_. For instance, NMR structures in *S*_1 _are refined by an appropriate X-ray structure *S*_2_. In this approach the different structural sources *S*_i _are usually not identical, as is evident in the case of solution and crystal structures, but they may differ also in other aspects such as in amino acid sequence or absence or presence of interacting molecules.

One important concept is that the available structural information from different sources is first converted into a dense network of derived substitute restraints *R*_i_^k* ^that can directly be compared (eq. 3). They are calculated from a structural bundle and are coded as main chain and side chain dihedral angle restraints, as well as distance restraints between selected sets of atoms. The expectation values and standard deviations *s *of the sample are directly calculated from the given structural bundle by the PERMOL-algorithm [23,24]. In case the leading structural set *S*_1 _consists of a set of NMR structures, such a bundle is already available. When no structural bundle is available, it first has to be created in a well-defined manner (see below). The restraints *R*_1_^k^* = *R*_1_^k ^(k = 1,..., M) are then combined with the sets of restraints *R*_i_^k^* (i = 2,...,N; k = 1,...,M_i_, M_i _≤ M) to obtain a final set of restraints *R*_0_^k ^(k = 1,..., M) and a new bundle of structures *S*_0 _is calculated. The quality of the new structural bundle can be validated against the original experimental data, a step which increases the confidence in the result and can be used to assess the improvement of the structures but is not required by the algorithm.

### Structure improvement of the Ras-binding domain of Byr2

As a first example, the AUREMOL-ISIC algorithm was tested on the structure improvement of the Ras-binding domain of Byr2 for which both a set of 10 solution NMR structures [16] and a single X-ray structure of Byr2 in complex with Ras [17] are available. The X-ray structure was used as source structure to improve the NMR structure *S*_1_.

**Figure 2:**
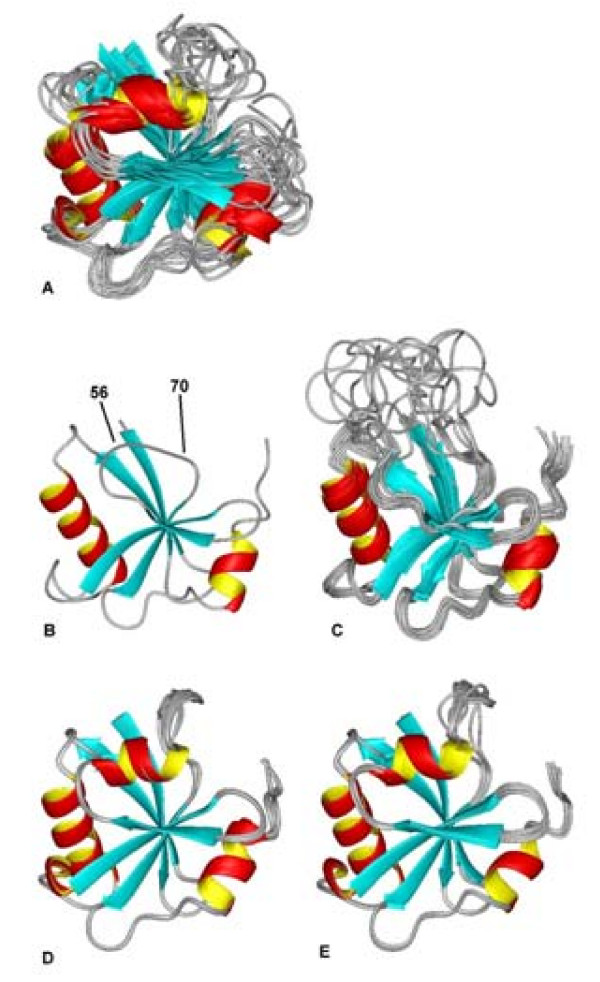
**Improvement of the solution structure of Byr2-RBD by an X-ray structure of the same molecule in complex with Ras**. Upper Panel: (A) NMR structural bundle of Byr2-RBD [16] [PDB ID: 1I35] (B) X-ray structure of Byr2-RBD in complex with Ras [17] [PDB ID: 1K8R]. Note that residues 57 – 69 could not be traced in the electron density map. (C) Structural bundle created from the X-ray structure by using the published resolution of 0.3 nm and the *B*-factors. Lower panel: (D) 10 final refined structures of Byr2-RBD without and (E) with refinement in explicit solvent.

As described above and using the parameters given in Table 1, distance and dihedral angle restraints were created that represent the X-ray data. In total 5248 distance restraints and 321 dihedral angle restraints were obtained, defining the restraint set *R*_2_^x^*. Please note that for residues 57 – 69 no restraints were obtained since these residues were invisible in the original X-ray structure. Employing these restraints and DYANA v.1.5 1000 structures were calculated. The 10 best in terms of DYANA target function were selected to define the set of structures *S*_2_^x ^that represents the X-ray data. For this purpose a standard DYANA simulated annealing protocol was used, which includes 4000 TAD (torsion angle dynamics) steps. One fifth of these are performed at an initial high temperature, followed by slow cooling during the rest of the schedule. Figures 2B and 2C show a comparison between the original X-ray structure and the corresponding set of structures *S*_2_^x^, respectively. As described above from the set *S*_2_^x ^the set of restraints *R*_2_* was generated. It consisted of 5600 distance restraints, 396 dihedral angle restraints and 53 hydrogen bond restraints. The corresponding parameters used for restraint generation are given in Table 2. The set of 10 submitted solution NMR structures defines the set of structures *S*_1 _(Fig. 2A), from which 6642 distance restraints, 453 dihedral angle restraints, and 106 hydrogen bond restraints were generated that define the leading restraint set *R*_1 _= *R*_1_*. Please note that 106 is the sum of all hydrogen bond restraints identified in the individual structures of the selected bundle. The corresponding parameters are given in Table 2. No separate structures were calculated using the restraint set *R*_1 _alone. In the next step the restraints from sets *R*_1_* and *R*_2_* were combined as described in the Materials and Methods section using the parameters given in Table 3. In the case of mismatching restraints only the restraint corresponding to the NMR structure was further used. After the restraint combination 6642 distance restraints, 338 dihedral angle restraints and 26 hydrogen bond restraints were obtained, defining the restraint set *R*_0_. Using the set *R*_0 _1000 structures were calculated with DYANA and the ten best in terms of the DYANA target function were selected for further analysis, defining the set *S*_0 _(Fig. 2D). The structures were refined in explicit solvent (water) [25,26]. As result a set (*S*_0_WR_) of 10 structures of Byr2-RBD (Fig. 2E) was obtained.

All secondary structure elements are well defined in these structures. Especially the C-terminal α-helix that was poorly characterized in the original NMR structures is now very well defined. In addition, the quality of the resulting structures was compared to the original NMR and X-ray structures (Table 4) employing rmsd calculations, Ramachandran plots, and NMR *R*-factor calculations. The results clearly show that the refined structures show improved values for all categories. The rmsd values of the newly calculated structures are drastically reduced compared to the original NMR structures, with values of 0.033 nm and 0.144 nm for the backbone N atoms, respectively. The percentage of residues in the most favored and allowed regions of the Ramachandran plot increased for the refined structures compared to both sets of input structures (*S*_1 _and *S*_2_). Since the goal was to obtain refined solution structures, the resulting structures have been analyzed, whether they really explain the experimental data better than the original structures. A suitable check for this purpose is the calculation of NMR *R*-factors [27] that directly compare an experimental NMR NOESY spectrum with the corresponding spectrum back-calculated from a single or a set of test structures. For the calculations shown in Table 4 we used the structurally most discriminating *R*-factor *R*_5 _as described by us previously [27]. The *R*-factors show also a significant improvement for the refined structures indicating that we were really able to obtain refined solution structures by the use of external data.

### Structure improvement of the Ras-binding domain of RalGDS-RBD

As a second test case the Ras-binding domain of RalGDS was chosen using a set of low resolution solution NMR structures as input together with a single X-ray structure of RalGDS in complex with Ras [19]. As in the first test case the X-ray structure was used to improve the NMR structure.

**Figure 3:**
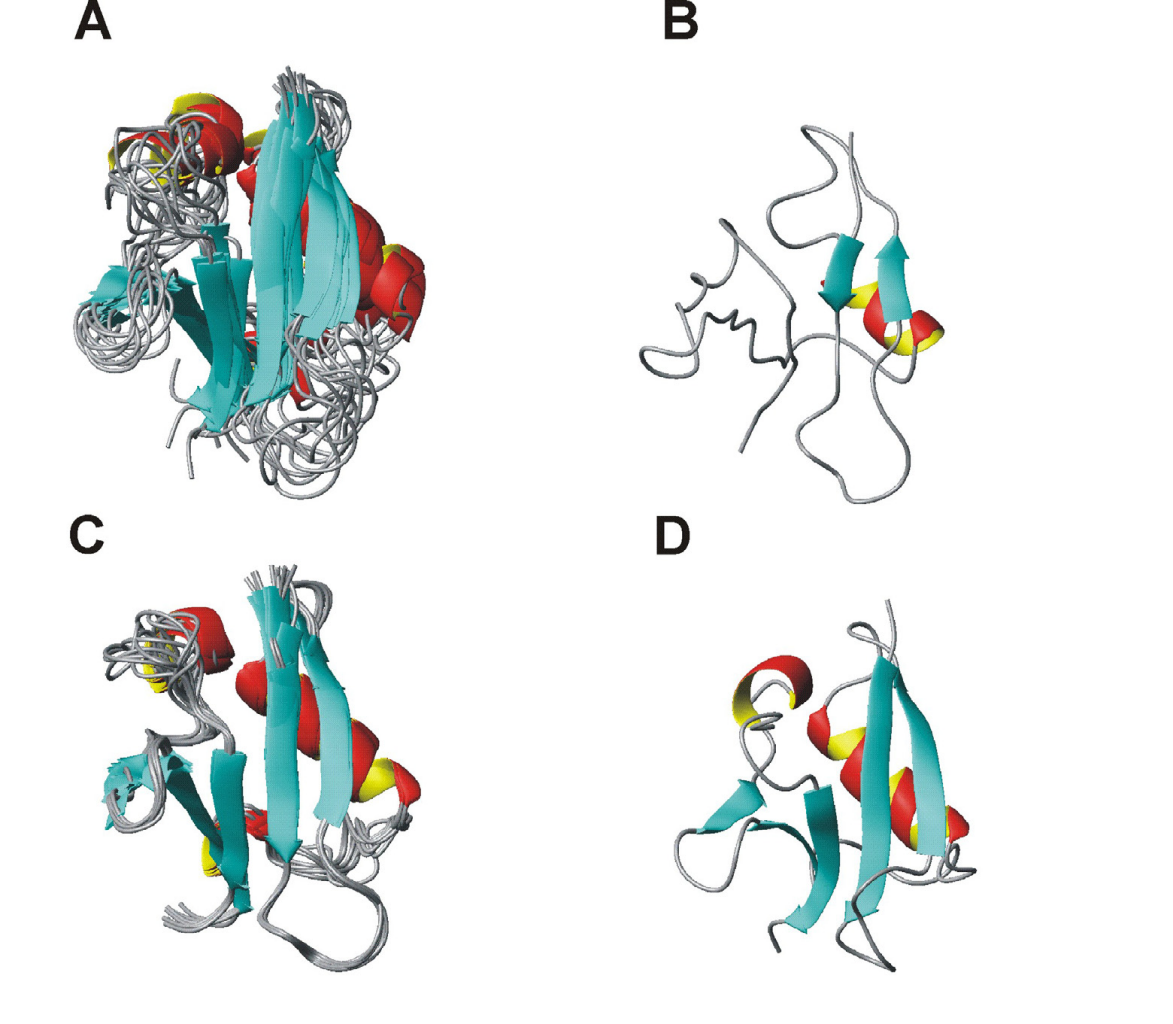
**Improvement of the solution structure of RalGDS-RBD by an X-ray structure of the same molecule in complex with Ras**. Upper Panel: (A) Newly calculated low resolution NMR structural bundle of RalGDS-RBD. (B) Input X-ray structure of RalGDS obtained in complex with Ras. Lower Panel: (C) 10 final refined structures of RalGDS-RBD without refinement in explicit solvent. (D) Reference NMR structure created from a full set of experimental restraints (25 h-bonds, 104 Φ and Ψ dihedral angles, and 1550 NOEs).

Low resolution NMR structures for RalGDS-RBD (residues 11–97) were newly calculated using easily available NMR data such as 25 h-bonds, 102 Φ and Ψ dihedral angles, and 232 backbone NOEs involving H_N_ and H_α_ atoms. Employing these restraints and DYANA v.1.5 300 structures were calculated as described above of which the 10 best in terms of DYANA target function were selected to define the set of NMR input structures *S*_1 _(Fig. 3A). As described above and using the parameters given in Table 5, distance and dihedral angle restraints were created that represent the X-ray data. In total 2001 distance restraints and 263 dihedral angle restraints were obtained, defining the restraint set *R*_2_^x^*. Please note that for residues 1, 50 – 55, 78 – 89, and 97 no restraints were obtained since these residues were invisible in the original X-ray structure. Employing these restraints and DYANA 1.5, 1000 structures were calculated, of which the 10 best in terms of DYANA target function were selected to define the set of structures *S*_2_^x ^that represents the X-ray data. The original input X-ray structure of RalGDS obtained in complex with Ras is shown in Figure 3B. As described above from the set *S*_2_^x ^the set of restraints *R*_2_* was generated consisting of 1784 distance restraints, 326 dihedral angle restraints and 13 hydrogen bond restraints. The corresponding parameters used for restraint generation are given in Table 6. The set of 10 low resolution NMR structures defines the set of structures *S*_1 _(Fig. 3A), from which 2344 distance restraints, 417 dihedral angle restraints, and 70 hydrogenbond restraints were generated that define the leading restraint set *R*_1 _= *R*_1_*. The corresponding parameters are given in Table 6. In the next step the restraints from sets *R*_1_* and *R*_2_* were combined as described in the Materials and Methods section using the parameters given in Table 7. In the case of mismatching restraints only the restraint corresponding to the NMR structure was further used. After restraint combination we obtained 2344 distance restraints, 285 dihedral angle restraints and 27 hydrogen bond restraints, defining the restraint set *R*_0_. Using the set *R*_0 _300 structures were calculated with DYANA and the ten best in terms of the DYANA target function were selected for further analysis, defining the set *S*_0 _(Fig. 3C). All secondary structure elements are well defined in these structures. Especially the locations of the two α-helices that were poorly defined in the input NMR structures are now substantially better defined. In addition, the quality of the resulting structures was compared to the original NMR structure (Fig 3D and Table 8) employing rmsd calculations, Ramachandran plots, and NMR *R*-factor calculations. The rmsd values of the newly calculated structures are drastically reduced compared to the input NMR structures with values of 0.07 nm and 0.21 nm for the rmsd values to the mean structure of the backbone N atoms, respectively. The corresponding average pair wise rmsd values for the backbone atoms show a similar trend with values of 0.11 nm and 0.33 nm, respectively (Table 8). This clearly shows the influence of the increased number of well defined restraints on the refined structures. The average pair wise rmsd difference between the low resolution NMR input structures and the refined structures amounts to 0.32 nm indicating on the one hand the influence of the second source (X-ray data) on the refinement and on the other hand that the refined structures are within the conformational space occupied by the low resolution NMR input structures. The percentage of residues in the most favored regions of the Ramachandran plot did not change for the refined structures compared to the low resolution input NMR structures (*S*_1_). The calculation of NMR R-factors was performed as described for Byr2-RBD. The *R*-factors show also a significant improvement for the refined structures indicating that we were able to obtain refined solution structures by the use of external data.

### Structure improvement of the B2 Immunoglobulin-Binding Domain of Streptococcal protein G

**Figure 4:**
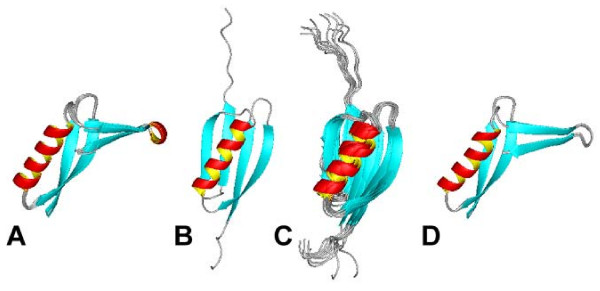
**Unbiased refinement of the solution structure of imunoglobulin binding domain of protein G**. Parameters used for restraint generation are the same as shown in Table 1 and Table 2. A: NMR structure (10 monomers of the dimeric structures). 2948 distance, 260 angle and 41 hydrogen bond restraints were obtained (*R*_1_*). The RMSD is 0.022 nm. The Ramachandran plot delivers 90% in most favored regions and 10% in additional allowed regions. B: Single monomeric X-ray structure. 1888 distance and 243 angle restraints were obtained for generation of the X-ray structure bundle (C). C: X-ray structure bundle (created as described in the materials and methods section). 2762 distance, 241 angle and 45 hydrogen bond restraints were obtained (*R*_2_*). D: 10 final refined structures obtained using the ISIC algorithm (restraint combination of A and C, 2948 distance, 224 angle and 26 hydrogen bond restraints were obtained). The RMSD is 0.008 nm for the backbone atoms. The Ramachandran plot delivers 92% in most favored regions and 8% in additional allowed regions.

The highest risk in using data from other sources to improve a target structure is a possible bias towards the added structure. In the worst case instead of a refined target structure the structure from the additional source is essentially reproduced. To investigate a possible bias introduced by an additional source on the ISIC algorithm two structures were selected, which clearly show different structural details. The solution structure of the B2 Immunoglobulin-Binding Domain of Streptococcal protein G [20] differs clearly from the X-ray structure [21]. The NMR structure was obtained from a dimeric form of the protein, where 4 core mutations lead to dimerization of the protein and a domain swapping of a β-pleated sheet. Figure 4A shows one half of the dimeric NMR structure compared the monomeric X-ray structure of the B2 domain (Fig. 4B). As it can clearly be seen the orientation of the last two β-strands is considerably different between the 2 structures. A simple averaging process between these two sets of structures leads to substantially incorrect structures and not to any improvements (data not shown). However, applying the ISIC algorithm however takes these structural differences automatically into account. We used the ISIC algorithm as described above by using the same parameters as described for Byr2-RBD and details of the calculations are given in the caption of figure 4. In the first step a bundle of structures representing the X-ray information (Fig. 4C) was generated. From this set and the NMR structures restraints were generated and combined with ISIC and new improved structures were calculated (Fig 4D). As can be seen from Figure 4D the resulting structures look very similar to the original NMR structure but the rmsd-values and the Ramachandran quality have slightly improved (Fig 4). Note that the original NMR structures were in this example already very well defined. We did also the inverse experiment, using the NMR-structure to improve the X-ray structure and obtained again an unbiased structure with all characteristics of the original structure (data not shown).

## Discussion and conclusion

Any determination of solution structures from experimental data is not (as sometimes automatically assumed) the direct calculation of the only existing solution but the search for a set of structures consistent with the experimental data and additional knowledge of the system (in this regard see also the paper by Rieping et al. [28]). The use of substitute restraints as introduced here with a simulated annealing protocol for restrained molecular dynamics is an efficient method to combine strongly coupled knowledge from different sources. A proper bias toward the selected target set of structures can be achieved by Bayesian reasoning, thus using the additional information only to increase the probability to find the "true" ground state set of structures corresponding to the experimental conditions selected. The combination with validation tools such as the calculation of NMR *R*-factors strengthens the impact of the method considerably since the improvement of the structures can be assessed quantitatively. This is clearly visible for the example of Byr2-RBD where our improved structures also better explain the experimental data. Even the choice of largely inappropriate additional knowledge does not lead to distortion of the original structure as shown for the immunoglobulin binding domain.

In the present paper the automated ISIC algorithm was used to improve a solution structure by related X-ray data. The qualities of both the originally submitted Byr2 NMR structures as well as the corresponding X-ray structure were both limited; therefore, giving an excellent example for testing the ISIC algorithm. The same is true for the RalGDS-RBD test case where both the set of low resolution NMR structures of RalGDS that were calculated only from easily available experimental data and the corresponding X-ray data are of medium quality. Especially this last test case is a good example how the inclusion of additional data can speed up the NMR structure determination process for example in structural genomics efforts. However, ISIC can also be used for other applications such as the improvement of a NMR structure of a given protein by NMR structures of homologues proteins or pure homology models. The same would be true for the improvement of X-ray structures by NMR-data when some parts of the electron density map are ill-defined.

Here, the X-ray R-factor would provide the validation tool. A similar application that one may encounter more often in the future is the calculation of NMR-structures of very large proteins using only a limited set of experimental data. One can think about other scenarios for the application of ISIC. When no X-ray structure of the protein is available homology models from related proteins may be used.

## Methods

### Details of the algorithm

#### Calculation of the network of substitute restraints

The calculation of a dense network of dihedral angle and distance restraints with the PERMOL-algorithm from bundles of structures has been described earlier [23,24]. and is implemented in AUREMOL [29]. Here, the expectation values and standard deviations are calculated. Error ranges are approximated from the standard deviations on the basis of the *t*-test. In case the original set contains only one structure the corresponding structural bundle has to be calculated first. In this regard we will discuss in the following only the most important case of crystal structures that are usually represented as distinct single structures *S*_i_^p ^(p = 1). But the principle can be applied to other data.

Depending on the unit cell and the refinement method used sometimes more than one structure is deposited in the data base (p > 1). However, even then the statistical ensemble is too small. The solution to this problem is that in analogy to the calculation of NMR-structures the inherent coordinate uncertainties can be used to calculate structural bundles and from those a set of substitute restraints *R*_i_* is obtained. Therefore, we first determine a set of restraints *R*_i_^x^* that represent the original X-ray structure(s) from inter-atomic distances and dihedral angles in the crystal structure(s) together with the corresponding coordinate uncertainties. Using these restraints a set of structures *S*_i_^x ^is created, from which the set of substitute restraints *R*_i_* is created using PERMOL. For generating the set *R*_i_^x^* two factors that are usually published together with the structure that can be used for a conservative estimate of the structural variations. In a first approximation the expected average error in atomic positions σ(r_0_) is about 1/3 of the resolution *R *[30]. In a more involved analysis σ(r_m_) of the atoms m possessing low *B*-factors is often estimated from Luzzati plots. Second the local *B*-factors can be used to introduce additional errors for specific atoms possessing significant *B*-values. Static and thermal disorder can effectively spread out the electron density of a given atom mand this increases its *B*-factor. The *B*-factor is related to the rms error in the position of an atom by the equation:

σ(rm)=Bm8⋅π2     (4)
 MathType@MTEF@5@5@+=feaafiart1ev1aaatCvAUfKttLearuWrP9MDH5MBPbIqV92AaeXatLxBI9gBaebbnrfifHhDYfgasaacH8akY=wiFfYdH8Gipec8Eeeu0xXdbba9frFj0=OqFfea0dXdd9vqai=hGuQ8kuc9pgc9s8qqaq=dirpe0xb9q8qiLsFr0=vr0=vr0dc8meaabaqaciaacaGaaeqabaqabeGadaaakeaaiiGacqWFdpWCcqGGOaakieqacqGFYbGCdaWgaaWcbaGaemyBa0gabeaakiabcMcaPiabg2da9maakaaabaWaaSaaaeaacqWGcbGqdaWgaaWcbaGaemyBa0gabeaaaOqaaiabiIda4iabgwSixlab=b8aWnaaCaaaleqabaGaeGOmaidaaaaaaeqaaOGaaCzcaiaaxMaadaqadaqaaiabisda0aGaayjkaiaawMcaaaaa@40EB@

*B*_m _denotes the *B*-factor of a given atom m and σ(**r**_m_) is the corresponding average error in atom positions.

Since for the calculations a conservative estimate of distances ranges is most useful, the square of the standard deviation σ^2^(*d*_*m*,*n*_) of the distance *d*_*m*,*n *_between two atoms m and n (m | n) is approximated by

σ^2^(*d*_*m*,*n*_) = σ(**r**_*m*_)^2 ^+ σ(**r**_*n*_)^2 ^+ 2σ(**r**_0_)^2 ^    (5)

For a more detailed description on the precision of protein structures see the article by Cruickshank [31]. When more than one structure of the same crystal is contained in the data base they can be considered as separate structural sets *S*_i _and handled in an analogous way. As mentioned above, using this preliminary set of restraints *R*_i_^x^* a bundle of structures *S*_i_^x ^is calculated by employing programs such as DYANA [32], XPLOR-NIH [33] or CNS [34]. From this bundle a set of restraints *R*_i_* is calculated in the same way as it has been done for the restraint set *R*_1 _of the leading structure *S*_1_.

#### Restraint combination

As derived above (eq. 2 and eq. 3), from the sets of restraints *R*_1 _(*R*_1 _= *R*_1_*) and *R*_i_* (i = 2,...,N) a new set *R*_0 _has to be calculated, which then enters then the final structure calculation. Although the algorithm produces restraint sets *R*_i_* that are matched to the leading set *R*_1 _for all data sets, in some cases no restraint *R*_i_^k* ^matching a restraint *R*_1_^k ^can be created for data set i. Such a case can occur when an atom or an amino acid of set *R*_1 _does not exist in the data used to generate set *R*_i_*. In this case *R*_0_^k ^is set to *R*_1_^k^. In all other cases the final restraint *R*_0_^k ^has to be calculated according to eq. 3. Since *P*(*R*_0_^k^|*R*_i_^k^*, i > 1) is difficult to determine for distances and angles, we apply a pair wise zero hypothesis test *P*(*R*_1_^k^|*R*_i_^k^*, i > 1), that the corresponding two restraints of the two data sets describe the same ensemble. If yes, a new probability distribution for the restraint is calculated, if no, the restraint *R*_i_^k^* is discarded and only *R*_1_^k ^is used. For the case that also errors in the leading restraint set *R*_1 _are expected it is possible to also discard the restraint *R*_1_^k^. However, this special option was not used in the current tests. When large structural bundles are created (as one of the possible options), the probability distributions can directly be obtained from the bundle. Since we have no *a priori *knowledge about the distribution type of the individual restraints, we can apply known statistical tests like the rank dispersion test according to Siegel and Tukey [35] or the comparison of two independent samples according to Kolmogoroff and Smirnoff [35]. In case that the investigated restraints possess the same or nearly the same type of distribution, the so called *U *test according to Wilcoxon, Mann and Whitney [35] can be applied. It is the distribution free counterpart to the parametrical Student *t*-test that strictly can only be applied for normally distributed data.

On a variety of data sets we tested according to Kolmogoroff and Smirnoff, whether our data can be assumed to follow a normal distribution. As a result it was found that for all our test cases the data are normally distributed within a small degree of error. Therefore, for practical reasons it is sufficient to assume that the distribution can be approximated sufficiently well by a Gaussian distribution.

As a consequence we are allowed to check for the null hypothesis by enforcing a pair-wise two-sided *t*-test that compares the individual distance and angle restraints of all restraint sets *R*_i_* (i > 1) with the corresponding restraints of set *R*_1_*. The average distances <dik*
 MathType@MTEF@5@5@+=feaafiart1ev1aaatCvAUfKttLearuWrP9MDH5MBPbIqV92AaeXatLxBI9gBaebbnrfifHhDYfgasaacH8akY=wiFfYdH8Gipec8Eeeu0xXdbba9frFj0=OqFfea0dXdd9vqai=hGuQ8kuc9pgc9s8qqaq=dirpe0xb9q8qiLsFr0=vr0=vr0dc8meaabaqaciaacaGaaeqabaqabeGadaaakeaacqWGKbazdaqhaaWcbaGaemyAaKgabaGaem4AaSMaeiOkaOcaaaaa@31C0@> and dihedral angles <aik*
 MathType@MTEF@5@5@+=feaafiart1ev1aaatCvAUfKttLearuWrP9MDH5MBPbIqV92AaeXatLxBI9gBaebbnrfifHhDYfgasaacH8akY=wiFfYdH8Gipec8Eeeu0xXdbba9frFj0=OqFfea0dXdd9vqai=hGuQ8kuc9pgc9s8qqaq=dirpe0xb9q8qiLsFr0=vr0=vr0dc8meaabaqaciaacaGaaeqabaqabeGadaaakeaacqWGHbqydaqhaaWcbaGaemyAaKgabaGaem4AaSMaeiOkaOcaaaaa@31BA@> together with the corresponding standard deviations s(*d*_i_^k*^) and s(*a*_i_^k*^) have been calculated from the structural bundles and the *t*-values *t*_1_^k ^(i > 1) are now calculated for the distances and angles by:

t1k=|<R1k>−<Rik*>|s2(R1k)L1+s2(Rik*)Li     (6)
 MathType@MTEF@5@5@+=feaafiart1ev1aaatCvAUfKttLearuWrP9MDH5MBPbIqV92AaeXatLxBI9gBaebbnrfifHhDYfgasaacH8akY=wiFfYdH8Gipec8Eeeu0xXdbba9frFj0=OqFfea0dXdd9vqai=hGuQ8kuc9pgc9s8qqaq=dirpe0xb9q8qiLsFr0=vr0=vr0dc8meaabaqaciaacaGaaeqabaqabeGadaaakeaacqWG0baDdaqhaaWcbaGaeGymaedabaGaem4AaSgaaOGaeyypa0ZaaSaaaeaadaabdaqaaiabgYda8iabdkfasnaaDaaaleaacqaIXaqmaeaacqWGRbWAaaGccqGH+aGpcqGHsislcqGH8aapcqWGsbGudaqhaaWcbaGaemyAaKgabaGaem4AaSMaeiOkaOcaaOGaeyOpa4dacaGLhWUaayjcSdaabaWaaOaaaeaadaWcaaqaaiabdohaZnaaCaaaleqabaGaeGOmaidaaOGaeiikaGIaemOuai1aa0baaSqaaiabigdaXaqaaiabdUgaRbaakiabcMcaPaqaaiabdYeamnaaBaaaleaacqaIXaqmaeqaaaaakiabgUcaRmaalaaabaGaem4Cam3aaWbaaSqabeaacqaIYaGmaaGccqGGOaakcqWGsbGudaqhaaWcbaGaemyAaKgabaGaem4AaSMaeiOkaOcaaOGaeiykaKcabaGaemitaW0aaSbaaSqaaiabdMgaPbqabaaaaaqabaaaaOGaaCzcaiaaxMaadaqadaqaaiabiAda2aGaayjkaiaawMcaaaaa@5DA0@

After that the individual *t*-values t1k
 MathType@MTEF@5@5@+=feaafiart1ev1aaatCvAUfKttLearuWrP9MDH5MBPbIqV92AaeXatLxBI9gBaebbnrfifHhDYfgasaacH8akY=wiFfYdH8Gipec8Eeeu0xXdbba9frFj0=OqFfea0dXdd9vqai=hGuQ8kuc9pgc9s8qqaq=dirpe0xb9q8qiLsFr0=vr0=vr0dc8meaabaqaciaacaGaaeqabaqabeGadaaakeaacqWG0baDdaqhaaWcbaGaeGymaedabaGaem4AaSgaaaaa@3099@ are compared to the critical *t*-value *t*_c_. The critical *t*-value at a given significance level and known degrees of freedom *f *(with *f *= *L*_1 _- *L*_*i *_- 1) can be calculated or looked up in the *t*-value table.

In case the calculated *t*-value *t*_1_^k ^is greater than the critical *t*-value *t*_c_, the null hypothesis has to be rejected and the restraint *R*_i_^k* ^is not used. Restraints with *t*_1_^k ^≤ *t*_c _are retained and the weighted average value <*R*_0_^k^> of the restraint *R*_0_^k ^is calculated together with the corresponding weighted total standard deviation σ(*R*_0_^k^).

#### Hydrogen bond restraints

In addition to combined dihedral angle and distance restraints the ISIC algorithm also uses backbone hydrogen bond restraints *R*_i_^k^. For the sake of clarity they will in the following be denoted as *H*_i_^k^. In principle hydrogen bonds could be handled in a similar way as described above for distance restraints by using the distributions of hydrogen bonding energies as parameters, where the hydrogen bond energies are calculated according to Freund [36]. Since rapid calculations are required within ISIC a somewhat faster method is actually used for hydrogen bond definition accepting a maximum NH-O distance of 0.24 nm and a hydrogen bond angle *a*_NHO _of 180° ± 35°. In ISIC the frequencies *X*_i_^k* ^of the hydrogen bonds in the different structural bundles *S*_i _are determined and used as hydrogen bond probabilities *P*(*H*_i_^k*^). From that the conditional probabilities *P*(*H*_0_^k^|*H*_1_^k^, *H*_i_^k*^, i = 2,...N) that a hydrogen bond exists in the solution structure are obtained.

P(H0k|H1k,Hik*,i=2,…,N)=P(H)(P(H1k,Hik*,i=1,…,N)P(H)(P(H1k,Hik*,i=2,…,N)+(1−P(H)(1−P(H1k,Hik,i=2,…,N))     (7)
 MathType@MTEF@5@5@+=feaafiart1ev1aaatCvAUfKttLearuWrP9MDH5MBPbIqV92AaeXatLxBI9gBaebbnrfifHhDYfgasaacH8akY=wiFfYdH8Gipec8Eeeu0xXdbba9frFj0=OqFfea0dXdd9vqai=hGuQ8kuc9pgc9s8qqaq=dirpe0xb9q8qiLsFr0=vr0=vr0dc8meaabaqaciaacaGaaeqabaqabeGadaaakeaacqWGqbaucqGGOaakcqWGibasdaqhaaWcbaGaeGimaadabaGaem4AaSgaaOGaeiiFaWNaemisaG0aa0baaSqaaiabigdaXaqaaiabdUgaRbaakiabcYcaSiabdIeainaaDaaaleaacqWGPbqAaeaacqWGRbWAaaGccqGGQaGkcqGGSaalcqWGPbqAcqGH9aqpcqaIYaGmcqGGSaalcqWIMaYscqGGSaalcqWGobGtcqGGPaqkcqGH9aqpdaWcaaqaaiabdcfaqjabcIcaOiabdIeaijabcMcaPiabcIcaOiabdcfaqjabcIcaOiabdIeainaaDaaaleaacqaIXaqmaeaacqWGRbWAaaGccqGGSaalcqWGibasdaqhaaWcbaGaemyAaKgabaGaem4AaSgaaOGaeiOkaOIaeiilaWIaemyAaKMaeyypa0JaeGymaeJaeiilaWIaeSOjGSKaeiilaWIaemOta4KaeiykaKcabaGaemiuaaLaeiikaGIaemisaGKaeiykaKIaeiikaGIaemiuaaLaeiikaGIaemisaG0aa0baaSqaaiabigdaXaqaaiabdUgaRbaakiabcYcaSiabdIeainaaDaaaleaacqWGPbqAaeaacqWGRbWAaaGccqGGQaGkcqGGSaalcqWGPbqAcqGH9aqpcqaIYaGmcqGGSaalcqWIMaYscqGGSaalcqWGobGtcqGGPaqkcqGHRaWkcqGGOaakcqaIXaqmcqGHsislcqWGqbaucqGGOaakcqWGibascqGGPaqkcqGGOaakcqaIXaqmcqGHsislcqWGqbaucqGGOaakcqWGibasdaqhaaWcbaGaeGymaedabaGaem4AaSgaaOGaeiilaWIaemisaG0aa0baaSqaaiabdMgaPbqaaiabdUgaRbaakiabcYcaSiabdMgaPjabg2da9iabikdaYiabcYcaSiablAciljabcYcaSiabd6eaojabcMcaPiabcMcaPaaacaWLjaGaaCzcamaabmaabaGaeG4naCdacaGLOaGaayzkaaaaaa@9C64@

Assuming that the restraints from different structural sets can be considered statistically independent and that with eq. 2 the probability *P*(*H*_i_^k^) that a hydrogen bond exists also under the conditions of true solution structures can be written as

*P*(*H*_i_^k^) = *P*(*H*_i_^k^|*H*_i_^k^*, i = 1,...,N)*P*(*H*_i_^k^*, i = 1,...,N)     (8)

one obtains from eq. 7 and eq. 8

P(H0k|H1k,Hik*,i=2,…,N)=P(H)(P(H1k⋅∏i=2NP(Hik|Hik*)P(Hik*)P(H)(P(H1k⋅∏i=2NP(Hik|Hik*)P(Hik*))+(1−P(H)(1−P(H0k)(P(H1k⋅∏i=2NP(Hik|Hik*)P(Hik*))     (9)
 MathType@MTEF@5@5@+=feaafiart1ev1aaatCvAUfKttLearuWrP9MDH5MBPbIqV92AaeXatLxBI9gBaebbnrfifHhDYfgasaacH8akY=wiFfYdH8Gipec8Eeeu0xXdbba9frFj0=OqFfea0dXdd9vqai=hGuQ8kuc9pgc9s8qqaq=dirpe0xb9q8qiLsFr0=vr0=vr0dc8meaabaqaciaacaGaaeqabaqabeGadaaakeaafaqaaeGabaaabaGaemiuaaLaeiikaGIaemisaG0aa0baaSqaaiabicdaWaqaaiabdUgaRbaakiabcYha8jabdIeainaaDaaaleaacqaIXaqmaeaacqWGRbWAaaGccqGGSaalcqWGibasdaqhaaWcbaGaemyAaKgabaGaem4AaSgaaOGaeiOkaOIaeiilaWIaemyAaKMaeyypa0JaeGOmaiJaeiilaWIaeSOjGSKaeiilaWIaemOta4KaeiykaKIaeyypa0dabaWaaSaaaeaacqWGqbaucqGGOaakcqWGibascqGGPaqkcqGGOaakcqWGqbaucqGGOaakcqWGibasdaqhaaWcbaGaeGymaedabaGaem4AaSgaaOGaeyyXIC9aaebCaeaacqWGqbaucqGGOaakcqWGibasdaqhaaWcbaGaemyAaKgabaGaem4AaSgaaOGaeiiFaWNaemisaG0aa0baaSqaaiabdMgaPbqaaiabdUgaRbaakiabcQcaQiabcMcaPiabdcfaqjabcIcaOiabdIeainaaDaaaleaacqWGPbqAaeaacqWGRbWAaaGccqGGQaGkcqGGPaqkaSqaaiabdMgaPjabg2da9iabikdaYaqaaiabd6eaobqdcqGHpis1aaGcbaGaemiuaaLaeiikaGIaemisaGKaeiykaKIaeiikaGIaemiuaaLaeiikaGIaemisaG0aa0baaSqaaiabigdaXaqaaiabdUgaRbaakiabgwSixpaarahabaGaemiuaaLaeiikaGIaemisaG0aa0baaSqaaiabdMgaPbqaaiabdUgaRbaakiabcYha8jabdIeainaaDaaaleaacqWGPbqAaeaacqWGRbWAaaGccqGGQaGkcqGGPaqkcqWGqbaucqGGOaakcqWGibasdaqhaaWcbaGaemyAaKgabaGaem4AaSgaaOGaeiOkaOIaeiykaKIaeiykaKIaey4kaSIaeiikaGIaeGymaeJaeyOeI0IaemiuaaLaeiikaGIaemisaGKaeiykaKIaeiikaGIaeGymaeJaeyOeI0IaemiuaaLaeiikaGIaemisaG0aa0baaSqaaiabicdaWaqaaiabdUgaRbaakiabcMcaPiabcIcaOiabdcfaqjabcIcaOiabdIeainaaDaaaleaacqaIXaqmaeaacqWGRbWAaaGccqGHflY1daqeWbqaaiabdcfaqjabcIcaOiabdIeainaaDaaaleaacqWGPbqAaeaacqWGRbWAaaGccqGG8baFcqWGibasdaqhaaWcbaGaemyAaKgabaGaem4AaSgaaOGaeiOkaOIaeiykaKIaemiuaaLaeiikaGIaemisaG0aa0baaSqaaiabdMgaPbqaaiabdUgaRbaakiabcQcaQiabcMcaPiabcMcaPaWcbaGaemyAaKMaeyypa0JaeGOmaidabaGaemOta4eaniabg+GivdaaleaacqWGPbqAcqGH9aqpcqaIYaGmaeaacqWGobGta0Gaey4dIunaaaaaaOGaaCzcaiaaxMaadaqadaqaaiabiMda5aGaayjkaiaawMcaaaaa@D2FF@

For the conditional probability that a hydrogen bond *P*(*H*_o_^k^|*H*_i_^k^*) also exists in solution when it exists in the crystal structure, a plausible value of 0.9 has been assumed in this paper. More accurate values for *P*(*H*_o_^k^|*H*_i_^k^*) could be obtained by a statistical analysis of the existing structural data base. The a priori probability *P*(*H*) that a hydrogen bond between a given pair of atoms exists is rather small, a plausible value would be 1/Q with Q the number of residues of the protein under consideration.

In case that *P*(H0k|H1k,Hik*
 MathType@MTEF@5@5@+=feaafiart1ev1aaatCvAUfKttLearuWrP9MDH5MBPbIqV92AaeXatLxBI9gBaebbnrfifHhDYfgasaacH8akY=wiFfYdH8Gipec8Eeeu0xXdbba9frFj0=OqFfea0dXdd9vqai=hGuQ8kuc9pgc9s8qqaq=dirpe0xb9q8qiLsFr0=vr0=vr0dc8meaabaqaciaacaGaaeqabaqabeGadaaakeaacqWGibasdaqhaaWcbaGaeGimaadabaGaem4AaSgaaOGaeiiFaWNaemisaG0aa0baaSqaaiabigdaXaqaaiabdUgaRbaakiabcYcaSiabdIeainaaDaaaleaacqWGPbqAaeaacqWGRbWAaaGccqGGQaGkaaa@3B2E@, *i *= 2,..., *N*) exceeds a given user-defined threshold, for example 0.75, the corresponding hydrogen bond restraint is accepted and transformed in appropriate distance restraints as usually done in MD-calculations.

#### Filtering of angle restraints

When dihedral angles are combined and averaged it is possible that the calculated average values are located in disallowed regions of the Ramachandran plot. A filter is implemented that allows the user to disregard backbone and side chain dihedral angles as a function of their presence in unfavorable regions of the Ramachandran plot.

### NMR spectroscopy and structures

The sequential assignments of the NMR signals of Byr2 and the experimental parameters have been described in [37]. A 2D ^1^H NOESY spectrum obtained with a mixing time of 100 ms was used for structure validation. As input data the NMR structure of the free Ras-binding domain of Byr2 (Byr2-RBD) from *Schizosaccharomyces pombe *(residues 71–165 here referred to as residues 1–95) [16] [PDB ID: 1I35], the crystal structure of Byr2-RBD in complex with Ras [17] [PDB ID: 1K8R], the NMR structure [20] [PDB ID: 1Q10] and the crystal structure [21] [PDB ID: 1PGX] of the immunoglobulin binding domain of protein G from *Streptococcus*, *species Lancefield group G *were selected.

### Programs and structure validation

NMR data evaluation was performed with the program AUREMOL (V 2.2.1). Expectation values and standard deviations of cyclic quantities were calculated according to Döker et al., [38]. Sequence alignment was performed with a module for pair-wise sequence alignment based on the Needleman-Wünsch algorithm and the BLOSUM62 matrix that we recently included in the AUREMOL module PERMOL [23,24]. The resulting refined solution structures were validated on the experimental NMR data by the calculation of NMR *R*-factors [27]. For investigating the stereo-chemical quality PROCHECK-NMR was employed [39] and rmsd values were calculated using MOLMOL [40].

### Molecular dynamics calculations

Structure calculations were performed using the torsion angle molecular dynamics program DYANA v1.5 [32]. Details of the used standard simulated annealing protocol are given in the corresponding publication. From the resulting structures the best in terms of DYANA target function were selected for refinement in explicit solvent [25,26].

### Implementation

ISIC is written in ANSI-C and is fully incorporated in the software package AUREMOL .

## Abbreviations

NMR, nuclear macgnetic resonance; rmsd, root mean square deviation; RBD, Ras binding domain.

## Authors' contributions

HRK, WG and KB conceived the project. KB and to a smaller part JMT wrote the ISIC software. KB, JMT and KPN implemented ISIC within the larger AUREMOL software package. KB calculated the improved structures and drafted the manuscript. WG and HRK coordinated the study and wrote the manuscript together with KB. All authors read and approved the final manuscript.

## Figures and Tables

**Table 1:** Permol parameter used for the generation of distance and angle restraints from out the X-ray structure (S_2_) which then are used in the MD calculation in order to create the X-ray bundle (S_2_^x^). Distances were calculated between every used atoms.

**Restraint generation parameter from the X-ray structure **(**S_2_**)	
Confidence level	99.00%
**Distances**	
Distance range	0.18 nm – 1.00 nm
Used atoms	N, C, C_α_, C_β_, C_γ_, C_δ_, C_ε_, C_ζ_, O
Number	5248
**Angles**	
Selected angles	ψ, φ, χ_1_, χ_2_, χ_21_, χ_22_, χ_3_, χ_31_, χ_32_, χ_4_, χ_5_, χ_6_
Number	321

**Table 2:** Permol parameters used for the generation of distance, angle and hydrogen bond restraints from the NMR Bundle (S_1_) and X-ray bundle (S_2_^x^) which then are used for combination.

Restraint generation parameter from the NMR Bundle (S_1_) and the X-ray Bundle (S_2_*) (*R*_2_)	
Confidence level	99.90%
Selected residues NMR	1–95
Selected residues X-ray	1–56, 70–95
Distance range bb	0.18 nm – 1.00 nm
Used Atoms bb	N, C
Distance range sc	0.18 nm – 0.60 nm
Used Atoms sc	H_N_, H_α_, H_α2_, H_α3_, H_β_, H_β1_, H_β2_, H_β3_, H_γ_, H_γ2_, H_γ3_, H_γ1_, H_δ_, H_δ1_, H_δ2_, H_δ3_, H_ε_, H_ε2_, H_ε3_, H_ε1_
Number NMR	6642
Number X-ray	5600
**Angles**	
Selected angles	ψ, φ, χ_1_, χ_2_, χ_21_, χ_22_, χ_3_, χ_31_, χ_32_, χ_4_, χ_5_, χ_6_
Number NMR	453
Number X-ray	396
**Hydrogen bonds**	
Donators	H_N_, H_γ_, H_η11_, H_η12_, H_η22_, H_ζ1_, H_ζ2_, H_ζ3_, H_γ1_
Acceptors	O, O_δ1_, O_δ2_, O_ε2_, N, N_η1_, N_η2_, N_δ2_
Number NMR	106
Number X-ray	53

**Table 3:** Restraint combination parameters and obtained numbers of restraints.

**Combination parameters**	
Angle filter	Favored regions, GLY, PRO, CHI1-CHI2: < level 2
H-bond threshold	0.75%
H-bond exchange	0.90%
Significance level	0.2%
**Number of obtained restraints**	
Distance	6642
Angles	338
H-bonds	26

**Table 4:** Quality values from AUREMOL and Procheck.

	**S**_**1**_**(NMR)**	**S**_**2**_**(X-ray)**	**S**_**0**_	**S**_**0_WR**_
AUREMOL R-fac (whole)	0.534	-	0.455	0.451
RMSD MolMol N [nm] to mean	0.144	0.067	0.026	0.033
Ramachandran m.f. + a. [%]	87.3	88.5	94.3	90.8
Most favored [%]	67.8	70.1	71.3	78.2
Additional allowed [%]	19.5	18.4	23.0	12.6
Generously allowed [%]	11.5	8.0	4.6	8.0
Disallowed [%]	1.1	3.4	1.1	1.1

**Table 5:** Permol parameter used for the generation of distance and angle restraints from out the X-ray structure (S_2_) which then are used in the MD calculation in order to create the X-ray bundle (S_2_^x^). Distances were calculated between every used atoms.

**Restraint generation parameter from the X-ray structure (S_2_) equal to TABLE 1**	
Confidence level	99.90%
**Distances**	
Distance range bb	0.18 nm – 1.00 nm
Used atoms	N, C, O
Distance range sc	0.18 nm – 1.00 nm
Used atoms	C_β_, C_γ_, C_δ_, C_ε_, C_ζ_
Number	2001
**Angles**	
Selected angles	ψ, φ, χ_1_, χ_2_, χ_21_, χ_22_, χ_3_, χ_31_, χ_32_, χ_4_, χ_5_, χ_6_
Number	263

**Table 6:** Permol parameters used for the generation of distance, angle and hydrogen bond restraints from the NMR Bundle (S_1_) and X-ray bundle (S_2_^x^) which then are used for combination.

Restraint generation parameter from the NMR Bundle (S_1_) and the X-ray Bundle (S_2_*) (*R*_2_)	
Confidence level	99.90%
Selected residues NMR	11–97
Selected residues X-ray	12–49, 56–77, 90–96
Distance range bb	0.18 nm – 1.00 nm
Used Atoms bb	N
Distance range sc	0.5 nm – 1.5 nm
Used Atoms sc	H_δ2_, H_δ21_, H_δ22_, H_δ3_, H_ε_, H_ε2_, H_ε3_, H_ε1_
Number NMR	2344
Number X-ray	1784
**Angles**	
Selected angles	ψ, φ, χ_1_, χ_2_, χ_21_, χ_22_, χ_3_, χ_31_, χ_32_, χ_4_, χ_5_, χ_6_
Number NMR	417
Number X-ray	326
**Hydrogen bonds**	
Donators	H_N_, H_γ_, H_η11_, H_η12_, H_η22_, H_ζ1_, H_ζ2_, H_ζ3_, H_γ1_
Acceptors	O, O_δ1_, O_δ2_, O_ε2_, N, N_η1_, N_η2_, N_δ2_
Number NMR	70
Number X-ray	13

**Table 7:** Restraint combination parameters and obtained numbers of restraints.

**Combination parameters**	
Angle filter	Favored regions, GLY, PRO, CHI1-CHI2: < level 2
H-bond threshold	0.75%
H-bond exchange	0.90%
Significance level	0.2%
**Number of obtained restraints**	
Distance	2344
Angles	285
H-bonds	27

**Table 8:** Quality values from AUREMOL and Procheck.

	**S_1_(NMR)**	**S_2_(X-ray)**	**S_0_**
AUREMOL R-fac (whole)	0.383	-	0.353
RMSD MolMol N [nm] to mean	0.21	0.13	0.07
RMSD MolMol bb [nm] pairwise	0.33	0.19	0.11
Ramachandran m.f. + a. [%]	91.3	74.4	88.8
Most favored [%]	72.8	36.7	72.8
Additional allowed [%]	18.5	38.0	16.0
Generously allowed [%]	6.2	16.5	7.4
Disallowed [%]	2.5	8.9	3.7
